# Aberrantly expressed long noncoding RNAs as potential prognostic biomarkers in newly diagnosed multiple myeloma: A systemic review and meta‐analysis

**DOI:** 10.1002/cam4.5135

**Published:** 2022-09-04

**Authors:** Jiading Qin, Bo Ke, Tingting Liu, Chunfang Kong, Anna Li, Huan Fu, Chenghao Jin

**Affiliations:** ^1^ Medical College of Nanchang University Nanchang Jiangxi 330006 China; ^2^ Department of Hematology Jiangxi Provincial People's Hospital Nanchang Jiangxi 330006 China; ^3^ National Clinical Research Center for Hematologic Diseases The First Affiliated Hospital of Soochow University Soochow Jiangsu 215006 China

**Keywords:** clinical prognosis, long noncoding RNA, meta‐analysis, multiple myeloma, predictive biomarker

## Abstract

**Background:**

Numerous studies have manifested long noncoding RNAs (lncRNAs) as biomarkers to determine the prognosis of multiple myeloma (MM) patients. Nevertheless, the prognostic role of lncRNAs in MM is still ambiguous. Herein, we performed a meta‐analysis to evaluate the predictive value of aberrantly expressed lncRNAs in MM.

**Methods:**

A systemic literature search was performed in PubMed, EMBASE, Cochrane, and Web of Science databases until October 9, 2021, and the protocol was registered in the PROSPERO database (CRD42021284364). Our study extracted the hazard ratios (HRs) and 95% confidence intervals (CIs) of overall survival (OS), progression‐free survival (PFS), or event‐free survival (EFS). Begg's and Egger's tests were employed to correct publication bias.

**Result:**

Twenty‐six individual studies containing 3501 MM patients were enrolled in this study. The results showed that aberrant expression of lncRNAs was associated with poor OS and PFS of MM patients. The pooled HRs for univariate OS and PFS were 1.48 (95% CI = 1.17–1.88, *p* < 0.001) and 1.30 (95% CI = 1.18‐1.43, *p* < 0.001), respectively, whereas the pooled HRs for multivariate OS and PFS were 1.50 (95% CI = 1.16‐1.95, *p* < 0.001) and 1.59 (95% CI = 1.22‐2.07, *p* < 0.001), respectively. Subgroup analysis suggested that MALAT1, TCF7, NEAT1, and PVT1 upregulation were associated with poor OS (*p* < 0.05), PVT1, and TCF7 upregulation were implicated with worse PFS (*p* < 0.05), while only TCF7 overexpression was correlated with reduced EFS (*p* < 0.05). Moreover, the contour‐enhanced funnel plot demonstrated the reliability of our current conclusion, which was not affected by publication bias.

**Conclusion:**

Aberrantly expressed particular lncRNAs are critical prognostic indicators in long‐term survival as well as promising biomarkers in progression‐free status. However, different cutoff values and dissimilar methods to assess lncRNA expression among studies may lead to heterogeneity.

## 1 INTRODUCTION

Multiple myeloma (MM) is the second most common incurable hematological disease of malignant plasma cells in the bone marrow, with an estimated prevalence of 12410 deaths in America in 2021.^1^ Chemotherapy remains the cornerstone for MM treatment, while the median survival time with chemotherapy is only about 3–5 years.^2^ The clinical disease course in patients with MM is very heterogeneous among individuals in survival outcomes, from just a few months to several years.^3^ Considering the significant discrepancy in prognosis, effective risk stratification deserves to be established. The revised‐International Staging System (R‐ISS) is regarded as the most commonly used prognostic factor in MM for risk stratification.^4^ However, patients within similar prognostic R‐ISS groups may manifest heterogeneous clinical outcomes, indicating that the current staging system is insufficient for stratifying patients with high‐risk features. To improve prognostic indicators and develop an individualized therapeutic strategy for MM patients, information regarding the molecular abnormalities driving these differences in results needs to be incorporated into medical care.^5^

LncRNAs are a class of noncoding RNAs with a length of longer than 200 nucleotides emerging as critical regulators for epigenetic, transcriptional, or posttranscriptional process.^6^ They participate in various regulations of the intracellular mechanism, including cell differentiation,^7^ transcription factor (TF) binding, histone modifications,^8^ and complex epigenomic patterns,^9^ leading to multiple biological functions alteration in MM cells, such as cell division, apoptosis, invasion, migration, immune response, and drug resistance.^10^, ^11^ Recently, accumulating studies have elaborated on the details that lncRNAs play a vital role in the pathogenesis of MM and affect its proliferation and apoptosis process.^12^, ^13^, ^14^ Numerous aberrantly expressed lncRNAs have been testified as potential prognostic indicators for MM patients, including prostate cancer‐associated transcript 1 (PCAT1),^15^ urothelial cancer‐associated 1 (UCA1),^16^ P53 regulation‐associated lncRNA (PRAL),^17^ antisense noncoding RNA in the INK4 locus (ANRIL),^18^, ^19^ and metastasis‐associated lung adenocarcinoma transcript 1 (MALAT1).^20^, ^21^ As declared by Allegra et al., circular RNA, a type of noncoding RNA, was abnormally expressed as multiple myeloma deteriorated,^22^ but the exact mechanism of abnormally expressed lncRNAs in the prognostic impact of MM is still unclear. Distinct clinical outcomes may occur due to the lack of multivariate analysis or the differences in sample size and study methods. Recent studies have shown that abnormally expressed lncRNA TCF7 can predict poor OS in MM patients,^23^, ^24^ but not the abnormally expressed lncRNA ANRIL,^18^ and the aberrant expression of lncRNA MALAT1 was not associated with clinical outcomes in MM patients,^25^ indicating the ambiguous prognostic significance of lncRNAs in MM. Meta‐analysis is considered an ideal statistical tool to eliminate the one‐sidedness of different sample sizes from individual studies. Hence, we implemented a meta‐analysis to elucidate the high‐risk group with poor prognoses and to further improve overall survival and recurrence incidence in the long term.

## 2 MATERIALS AND METHODS

### 2.1 Study search strategy

We conducted this systematic review and meta‐analysis according to the requirements of Preferred Reporting Items for Systematic Reviews and Meta‐analysis (PRISMA) reporting guideline.^26^ The protocol of this study was preregistered in the PROSPERO database (CRD42021284364).

A systemic literature search was performed in PubMed, EMBASE, Cochrane, and Web of Science databases until October 9, 2021 with no beginning search date and language limitations. The reference list of relevant articles was then checked. The specific search formula is as follows: (“Multiple Myeloma” OR “Multiple Myelomas” OR “Plasma‐Cell Myeloma” OR “Plasma‐Cell Myelomas” OR “Myelomatosis” OR “Myelomatoses” OR “Plasma Cell Myeloma” OR “Plasma Cell Myelomas” OR “Kahler Disease” OR “Myeloma‐Multiple” OR “Myeloma Multiple” OR “Myeloma‐Multiples”) AND (“RNA, Long Noncoding” OR “lncRNA” OR “Long ncRNA” OR “Long Non‐Translated RNA” OR “Long Non‐coding RNA” OR “Long Non Coding RNA” OR “Long Non‐Protein‐Coding RNA” OR “Long Non Protein Coding RNA” OR “Long Non‐coding RNA” OR “Long Untranslated RNA” OR “Long ncRNAs” OR “Long Intergenic Non‐Protein Coding RNA” OR “Long Intergenic Non Protein Coding RNA” OR “LincRNAs” OR “LINC RNA” OR “LincRNA”) AND (“Prognosis” OR “Prognoses” OR “Prognostic Factors” OR “Prognostic Factor”).

### 2.2 Inclusion and exclusion criteria

Studies were included according to the following criteria: (1) diagnosed with de novo MM, (2) analyzed the association between MM and lncRNAs, (3) established a specific and clear cutoff to distinguish lncRNAs expression group rather than risk scores, (4) usable or sufficient survival data were given to extract or calculate 95% CIs of HR, (5) multivariate or univariate proportional hazard models adjusted for primary prognostic values such as overall survival (OS), progression‐free survival (PFS), or event‐free survival (EFS) were enrolled in statistical analyses. Letters, reviews, case reports, conference abstracts, non‐English literature, and retracted papers were excluded. Paper selection and data extraction were independently conducted by two authors (JDQ and BK). Endnote software (version 18.0) was used to exclude duplicates from different bibliographic databases to select eligible studies. JDQ, TTL, and CHJ participated in discussions to resolve any discrepancy in determining included studies.

### 2.3 Data extraction

Information including the publication year, first author, lncRNAs, country, ethnicity, sample type, method, the total number of cases, sex, follow‐up months, the cutoff value, ISS stage, serotype, study design, and outcomes were extracted by two authors (JDQ and HF). OS, PFS, or EFS was regarded as a prognostic endpoint for this meta‐analysis, and the hazard ratios (HRs) and 95% CIs were directly extracted from the studies. Univariate HRs were obtained from the given Kaplan–Meier survival curves using the Engauge Digitizer (version 4.1) if the HRs of OS, PFS, or EFS were not mentioned in the articles.^27^ JDQ and ANL evaluated the methodological quality of all included literature using the Newcastle‐Ottawa quality assessment scale (NOS) for cohort studies independently.^28^ NOS was divided into three criteria: selection, comparability, and outcome, which could be given a maximum of 4, 2, and 3 stars, respectively, according to the nine dimensions. Literature with a final score of no less than six stars was regarded as a high‐quality article.

### 2.4 Statistical analysis

All the data were analyzed using STATA software (version 16.0, Stata Corp. College Station, TX, USA). The HRs and 95% CIs measured the association between MM prognosis and lncRNA expression levels. Cochran's Q test and *I* ^2^ statistics were performed to evaluate statistical heterogeneity between studies. If significant heterogeneity existed between studies (*I* ^2^ > 50% or *p* < 0.05), a random effect model was used, yet if there was no significant heterogeneity between studies (*I* ^2^ ≤ 50% or *p* ≥ 0.05), a fixed effect model was utilized. Publication bias that may be somewhat ascribed to the inclination for positive results was assessed using Egger's and Begg's tests, and the two‐tailed *p* value of less than 0.05 was considered to have publication bias.^29^, ^30^ Trim and fill analysis of Duval and Tweedie was then used to evaluate the number of missing studies. We recalculated the pooled odds ratio by adding those missing hypothetical studies.^31^ In addition, heterogeneity was analyzed to evaluate the reliability of our conclusion using *I* ^2^ and *τ* ^2^, and publication bias was visualized using a contour‐enhanced funnel plot according to the procedures as previous.^32^

## 3 RESULTS

### 3.1 Study selection

**FIGURE 1:**
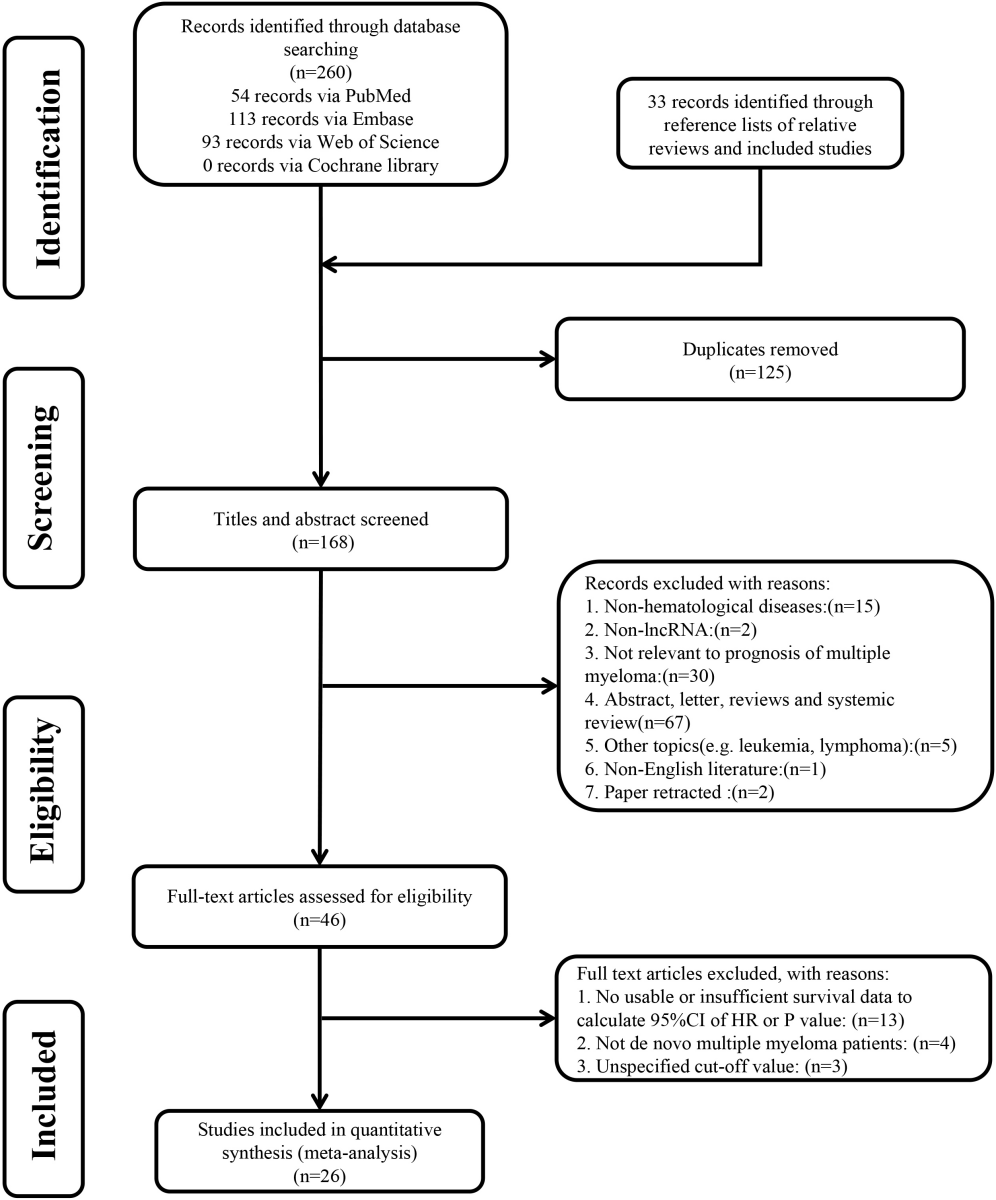
The PRISMA flow diagram

As shown in Figure 1. a total of 293 articles were retrieved, and 168 abstracts were screened after excluding duplicate reports. The included studies were then screened by title and abstract, followed by a full‐text review. Ultimately, 26 articles that met the criteria were included in the final analysis.^14^, ^17^, ^18^, ^23^, ^24^, ^33^, ^34^, ^35^, ^36^, ^37^, ^38^, ^39^, ^40^, ^41^, ^42^, ^43^, ^44^, ^45^, ^46^, ^47^, ^48^, ^49^, ^50^, ^51^, ^52^, ^53^

### 3.2 Characteristics of included studies

Twenty‐six cohort studies, containing 3501 de novo MM patients, were enrolled in this meta‐analysis and their characteristics are listed in Table 1. All of the studies were published between 2014 and 2021, comprising 20 different lncRNAs. LncRNA TCF7 and NEAT1 have been reported in three articles, two of which referred to PVT1, NEAT1, and MALAT1. Among these 26 studies, 24 studies used bone marrow aspirations to obtain the clinical samples, and 2 studies used peripheral fasting venous blood. Asian accounts for the majority of patients except for five studies, 19 studies used the median value as the cutoff threshold to distinguish high expression and low expression, 24 records reported OS, 11 reported PFS, and only 3 reported EFS. Twelve records were prospective cohort studies, whereas the others were retrospective cohort studies.

**TABLE 1:** Basic information of included studies and lncRNA expression‐related patients

Study cohort	Country	Ethnicity	LncRNA	T	Method	No. (M/F)	Follow‐up	ISS stage			Serotype			Cutoff	Outcome	HR
								I	II	III	IgG IgA Other					
Cho SF (2014)	China	Asian	MALAT1	B	qRT‐PCR	45 (29/16)	21 (2–48)	7	17	21	21	13	11	3.5a	PFS/OS	P(U)
Sedlarikova L(2017)	Czech Republic	Caucasian	UCA1; NEAT1	B	qRT‐PCR	84 (40/44)	27 (1–63)	23	26	35	55	18	11	4.313; 0.609	OS	R(M/U)
Xiao G (2018)	China	Asian	PRAL	B	qRT‐PCR	42 (23/19)	20 (1–60)	14	20	8	16	7	19	Median	DFS/OS	P(M/U)
Dong H (2019)	China	Asian	ST3GAL6‐AS1	B	qRT‐PCR	86 (53/33)	27.5 (2–61)	16	33	37	43	22	21	Median	PFS	P(U)
Li P (2020)	China	Asian	PVT1	B	qRT‐PCR	128 (82/46)	21.5 (1–42)	31	33	64	70	35	23	Median	PFS/OS	P(M/U)
Wang Y (2020)	China	Asian	OIP5‐AS1	B	qRT‐PCR	38 (18/20)	21 (1–50)	8	14	16	18	12	8	Median	OS	P(U)
Yu H (2020)	China	Asian	NEAT1	B	qRT‐PCR	114 (68/46)	20 (1–42)	29	27	58	62	27	25	Median	PFS/OS	P(U)
Zhang C (2020)	China	Asian	TCF7	B	qRT‐PCR	216 (133/83)	29 (2–47)	44	61	111	125	48	43	Median	PFS/OS	P(U)
Handa H (2020)	Japan	Asian	PVT1	B	qRT‐PCR	204 (164/40)	23 (1–66)	*	*	*	*	*	*	Upper quartile	PFS/OS	P(M/U)
Zhao P (2021)	China	Asian	PCAT1	B	qRT‐PCR	83 (51/32)	26 (1–48)	12	28	43	47	15	21	Median	PFS/OS	R(M/U)
Yin Y (2021)	China	Asian	ANRIL	B	qRT‐PCR	87 (52/35)	23 (2–42)	22	19	46	47	21	19	Median	PFS/OS	P(M/U)
Ding T (2021)	China	Asian	TCF7	B	qRT‐PCR	132 (82/50)	25 (2–36)	35	45	52	73	30	29	Median	EFS/OS	P(U)
He X (2021)	China	Asian	LINC01606	V	qRT‐PCR	72	20 (2–36)	*	*	*	*	*	*	3.495	OS	P(U)
TodoertiK (2021)	Italy	Caucasian	SNHG6	B	Illumina	497	30.2 (1–66)	155	177	165	*	*	*	Median	PFS/OS	R(M/U)
Liu H (2021)	China	Asian	TCF7	B	qRT‐PCR	86 (53/33)	29 (3–43)	19	32	35	49	20	17	Median	EFS/OS	P(M/U)
Wang Y (2020)	China	Asian	H19	V	qRT‐PCR	60	22 (2–50)	*	*	*	*	*	*	Y#	OS	R(U)
Li F (2020)	China	Asian	CRNDE	B	qRT‐PCR	80 (42/38)	1–48	*	*	*	*	*	*	Median	PFS/OS	R(M)
David A (2020)	France	Caucasian	CRNDE	B	qRT‐PCR	70	57 (3–250)	*	*	*	*	*	*	Median	OS	R(U)
Zhang Y(2020)	China	Asian	TUG1	B	qRT‐PCR	49 (26/23)	23 (1–60)	8	21	20	*	*	*	Median	OS	R(U)
Zhang W (2020)	China	Caucasian	EPB41L4A	B	Affymetrix	556 (334/222)	36 (1–100)	295	145	116	311	133	112	M#	EFS/OS	R(U)
Huang L (2019)	China	Asian	SNHG18 SEMA5A	B	qRT‐PCR	36 (17/19)	15 (1–33)	16	6	14	17	10	9	Median	OS	R(U)
Nian F (2019)	China	Asian	ANGPTL1‐3	B	qRT‐PCR	36	31 (5–60)	*	*	*	*	*	*	Median	OS	R(U)
Handa H (2019)	Japan	Asian	MALAT1	B	qRT‐PCR	77 (42/35)	27 (1–27)	12	39	26	47	16	14	Median	PFS/OS	R(U)
Wu Y (2017)	China	Asian	NEAT1	B	qRT‐PCR	51 (14/37)	19 (1–50)	20	16	15	*	*	*	Y#	OS	R(U)
Sun Y (2017)	China	Asian	H19	B	qRT‐PCR	30 (17/13)	26 (1–60)	*	*	*	*	*	*	Median	DFS	R(U)
Leon A (2020)	Spain	Caucasian	PDLIM1P4	B	Illumina	542	25 (1–66)	*	*	*	*	*	*	Median	PFS/OS	R(U)

Abbreviations: B, bone marrow; DFS, disease‐free survival; HR, hazard ratio; lncRNA, long noncoding RNA; M#, maximally selected rank statistics; OS, overall survival; PFS, progression‐free survival; qRT‐PCR, quantitative reverse transcription‐polymerase chain reaction; T, tissue; V, venous blood; *, NA; Y#, Youden index.

### 3.3 The quality assessment

We performed NOS to assess the quality of these articles. Among these 26 studies, 6 articles received nine stars, 12 received eight stars, 6 received seven stars, and 2 received six stars, suggesting the high quality of these included articles. The detailed scores are displayed in Table 2.

**TABLE 2:** Quality assessment of the individual study

Study cohort	Cohort selection criteria				Comparability	Study assessment			Score
	Representativeness of exposed cohort	Selection of nonexposed cohort	Ascertainment of exposure	Outcome not present at the start		Assessment of outcome	Follow‐up length	Follow‐up adequacy	
Cho SF (2014)	*	*	*	*	*	*	*	*	8
Sedlarikova L (2017)	*	*	*	*	**	*	*	*	9
Xiao G (2018)	*	*	*	*	**	*	*	*	9
Dong H (2019)	*	*	*	*	*	*	*	*	8
Li P (2020)	*	*	*	*	*	*	*	*	8
Wang Y (2020)	*	*	*	*	*	*	*	*	8
Yu H (2020)	*	*	*	*	—	*	*	*	7
Zhang C (2020)	*	*	*	*	—	*	*	*	7
Handa H (2020)	*	*	*	*	*	*	*	*	8
Zhao P (2021)	*	*	*	*	**	*	*	*	9
Yin Y (2021)	*	*	*	*	**	*	*	*	9
Ding T (2021)	*	*	*	*	*	*	—	*	7
He X (2021)	*	*	*	*	*	*	—	*	7
Todoerti K (2021)	*	*	*	*	*	*	*	*	8
Liu H (2021)	*	*	*	*	**	*	*	*	9
Wang Y (2020)	*	*	*	*	—	*	—	*	6
Li F (2020)	*	*	*	*	*	*	*	*	8
David A (2020)	*	*	*	*	—	*	*	*	7
Zhang Y (2020)	*	*	*	*	*	*	*	*	8
Zhang W (2020)	*	*	*	*	*	*	*	*	8
Huang L (2019)	*	*	*	*	**	*	*	—	8
Nian F (2019)	*	*	*	*	—	*	—	*	6
Handa H (2019)	*	*	*	*	**	*	*	—	8
Wu Y (2017)	*	*	*	*	**	*	*	*	9
Sun Y (2017)	*	*	*	*	—	*	*	*	7
Leon A (2020)	*	*	*	*	*	*	*	*	8

*Note*: For comparability, if the multivariate analysis is used to correct age‐mixed factors, two asterisks are obtained. If the age is not fixed and there is no significant difference in age, ISS stage, etc., an asterisk is given; otherwise, 0 asterisks are obtained. For follow‐up adequacy, if the rate of loss to follow‐up is <20%, an asterisk is scored. If the rate of loss to follow‐up is not described or ≥20%, 0 asterisks are obtained.

### 3.4 Analysis of outcome

**FIGURE 2:**
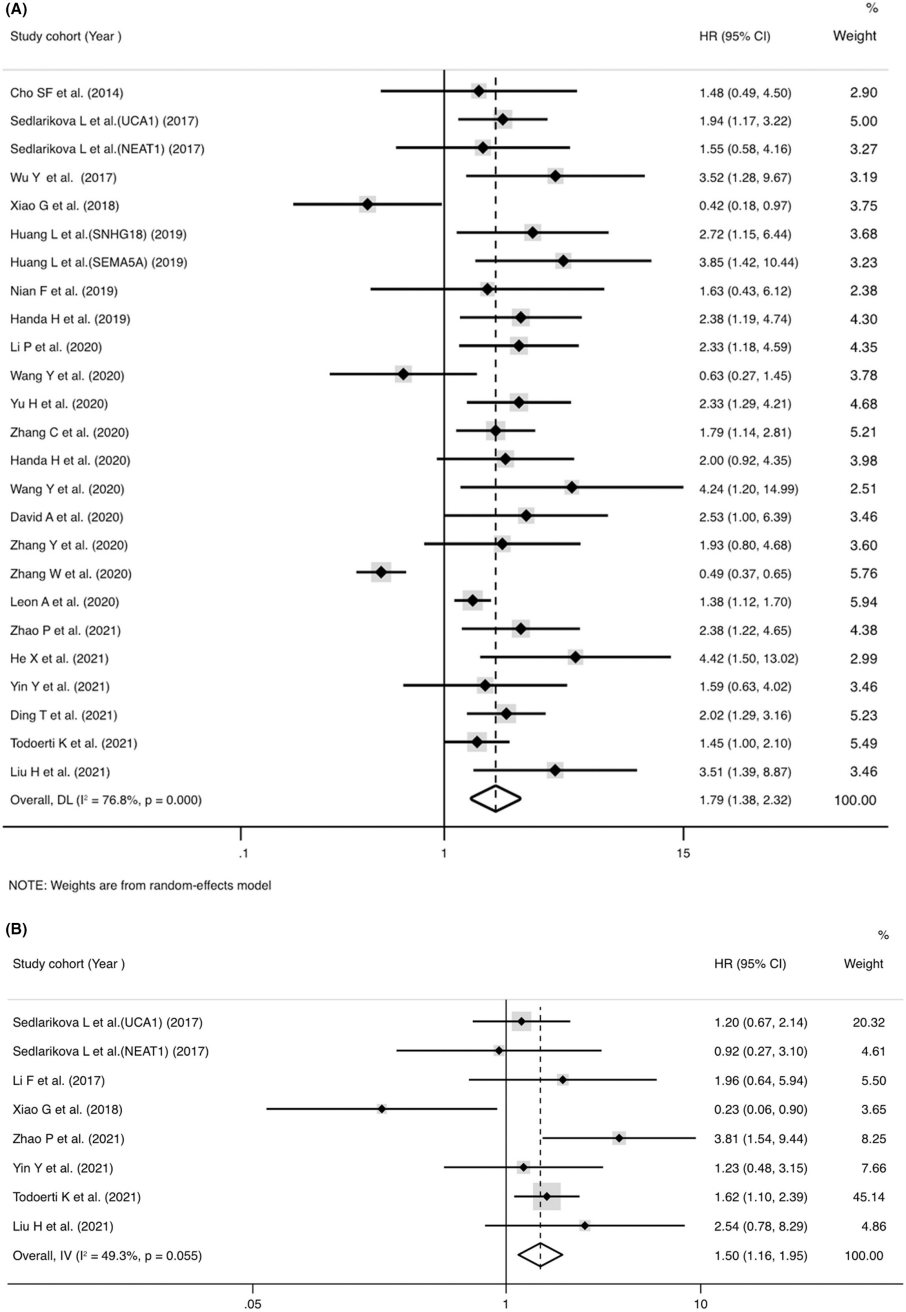
Forest plot of pooled hazard ratios (HRs) and 95% confidence intervals to access the prognostic value of long noncoding RNAs in overall survival (OS) of de novo multiple myeloma patients. (A) Univariate HRs of OS and (B) multivariate HRs of OS.

**FIGURE 3:**
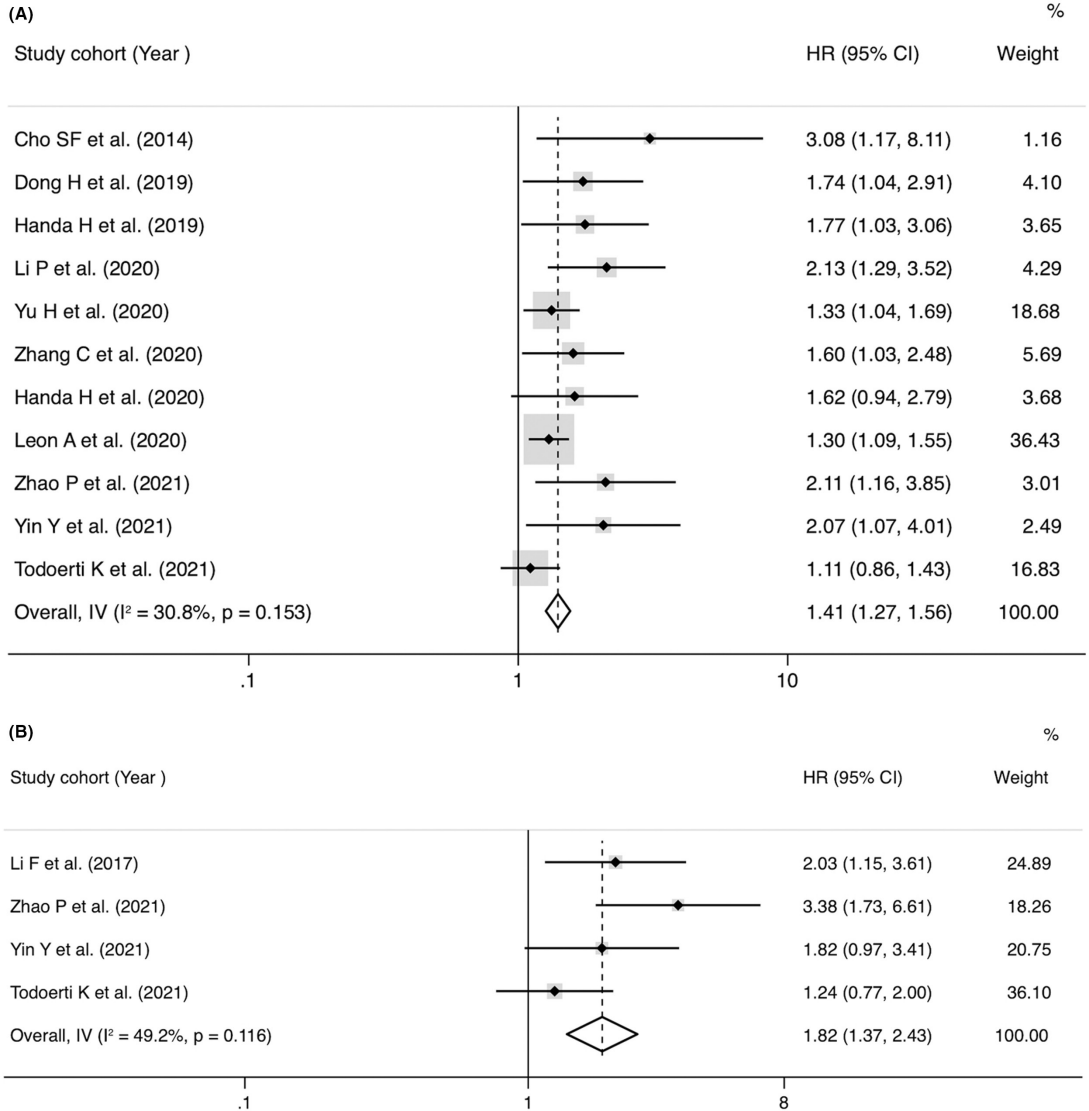
Forest plot of pooled hazard ratios (HRs) and 95% confidence intervals to access the prognostic value of long noncoding RNAs in progression‐free survival (PFS) of de novo multiple myeloma patients. (A) Univariate HRs of PFS and (B) multivariate HRs of PFS.

Twenty‐three studies mentioned the association between lncRNAs and OS assessed by univariate analysis (Figure 2A), of which 7 reported HRs of OS in multivariate analysis (Figure 2B). The abnormally overexpressed lncRNAs are concerned with worse prognosis in OS of MM patients with univariate analysis (pooled HR = 1.79, 95% CI = 1.38‐2.32, *p* < 0.001, *I* ^2^ = 76.8%, random effect) or multivariate analysis (pooled HR = 1.50, 95% CI = 1.16–1.95, *p* = 0.002, *I* ^2^ = 49.3%, fixed effect). Eleven studies recorded the correction between lncRNAs and PFS evaluated by univariate analysis (Figure 3A), of which four reported HRs of PFS in multivariate analysis (Figure 3B). The abnormally overexpressed lncRNAs are concerned with worse prognosis in PFS of MM patients with univariate analysis (HR = 1.40, 95% CI = 1.26–1.56, *p* < 0.001, *I* ^2^ = 30.8%, fixed effect) or multivariate analysis (HR = 1.82, 95% CI = 1.37‐2.42, *p* < 0.001, *I* ^2^ = 49.2%, random effect).

### 3.5 Subgroup analysis and heterogeneity exploration

**FIGURE 4:**
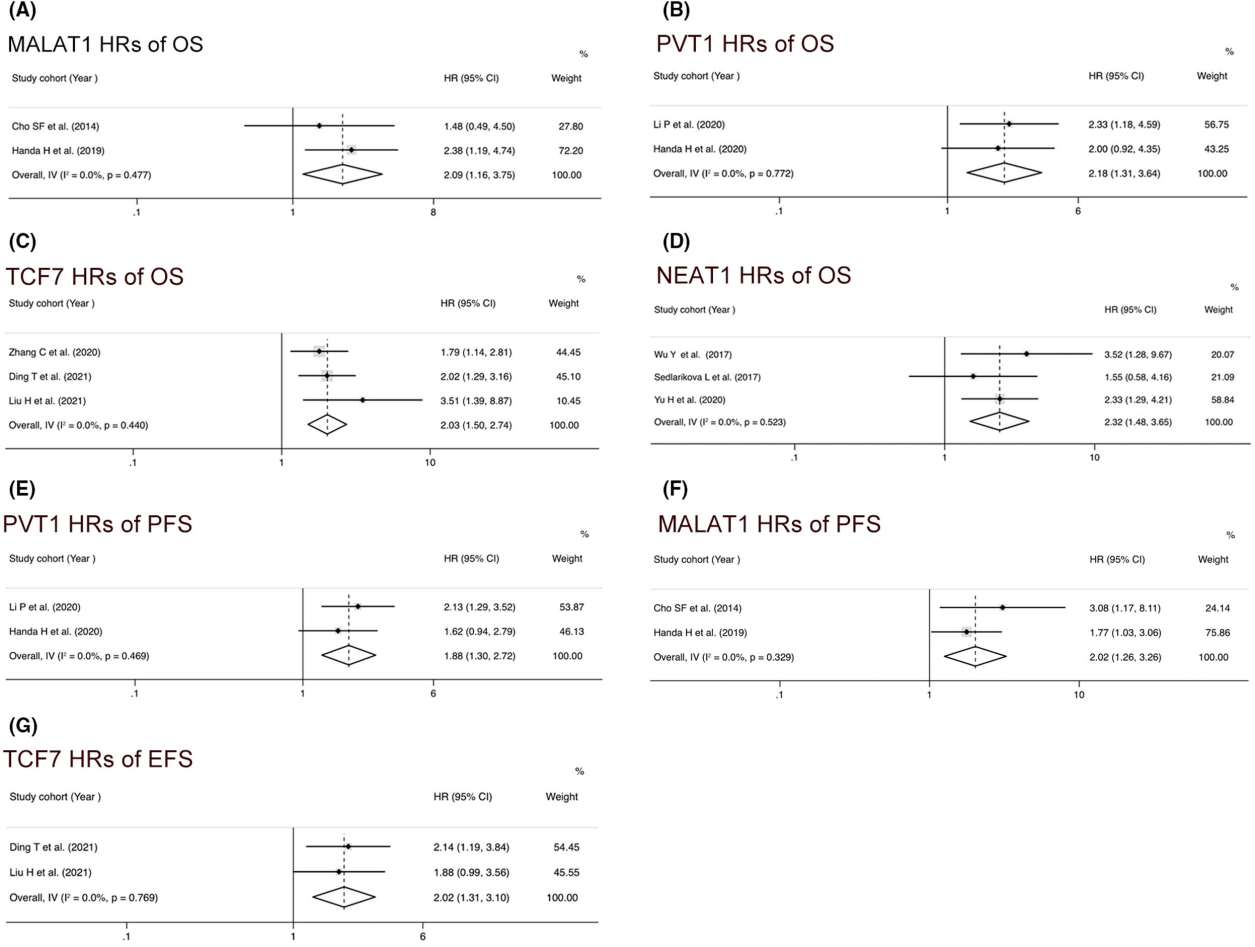
Forest plot of pooled hazard ratios (HRs) and 95% confidence intervals for multiple myeloma patients to access the prognostic value of different long noncoding RNAs. (A) MALAT1 HRs of overall survival (OS), (B) PVT1 HRs of OS, (C) TCF7 HRs of OS, (D) NEAT1 HRs of OS, (E) PVT1 HRs of PFS, (F) MALAT1 HRs of progression‐free survival, and (G) TCF7 HRs of event‐free survival.

MALAT1, TCF7, PVT1, and NEAT1 have been analyzed in two or more studies, some of which reported not only OS but also PFS or EFS. MALAT1 overexpression was associated with worse OS (HR = 2.09, 95% CI = 1.16–3.75, *p* = 0.014, *I* ^2^ = 0, fixed effect) (Figure 4A) and worse PFS (HR = 2.02, 95% CI = 1.26–3.26, *p* = 0.001, *I* ^2^ = 0, fixed effect) (Figure 4F). PVT1 upregulation was associated with worse OS (HR = 2.18, 95% CI = 1.31–3.64, *p* = 0.003, *I* ^2^ = 0, fixed effect) (Figure 4B) and poor PFS (HR = 1.88, 95% CI = 1.30–2.72, *p* = 0.001, *I* ^2^ = 0, fixed effect) (Figure 4E). TCF7 upregulation was correlated with worse OS (HR = 2.03, 95% CI = 1.50–2.74, *p* < 0.001, *I* ^2^ = 0, fixed effect) (Figure 4C) and poor EFS (HR = 2.02, 95% CI = 1.31–3.10, *p* = 0.001, *I* ^2^ = 0, fixed effect) (Figure 4G), respectively. We also observed that upregulation of NEAT1 were correlated with worse OS (HR = 2.32, 95% CI = 1.48–3.65, *p* = 0.004, *I* ^2^ = 0, fixed effect) (Figure 4D).

To further explore the correction between lncRNAs expression and the OS or PFS, we classified included patients based on analysis type, ethnicity, sample sizes, lncRNAs, maximum follow‐up time, ISS stage, serotype, the methods of assessing lncRNA expression, and cutoff value. This study performed the subgroup analysis of OS in two or more studies. As shown in Table 3, we noticed a significantly worse OS in MM patients with abnormal overexpression in both groups of sample size < 100 (HR = 1.93, 95% CI = 1.42–2.62, *p* = 0.017) and sample size ≥100 (HR = 1.63, 95% CI = 1.09–2.44, *p* = 0.019). Similarly, the shorter OS existed in subgroups of maximum follow‐up ≥5 years (HR = 1.88, 95% CI = 1.27–2.80, *p* = 0.002) and follow‐up <5 years (HR = 1.74, 95% CI = 1.24–2.44, *p =* 0.001). A strong correction was discovered between abnormal lncRNAs expression and poor OS in study cohort with ISS stage I >25% (HR = 1.86, 95% CI = 1.01–3.44, *p =* 0.049) but not with ISS stage I ≤25% (HR = 1.57, 95% CI = 0.98–2.53, *p* = 0.062). Simultaneously, the combined HRs of abnormal lncRNAs expression on OS in study cohort with serotype IgG >55% and serotype IgG ≤55% were 1.74 (95% CI = 0.98–3.07, *p* = 0.058) and 1.57 (95% CI = 0.94–2.62, *p* = 0.088), respectively. Similarly, the combined HRs of abnormal lncRNAs expression on OS in study cohort with serotype IgA >25% and serotype IgA ≤25% were 1.86 (95% CI = 1.01–3.44, *p* = 0.049) and 1.57 (95% CI = 0.98–2.53, *p* = 0.062), respectively. Furthermore, in terms of ethnicity, sample sizes, maximum follow‐up time, ISS stage, serotype, and cutoff values, we observed significant heterogeneity in these subgroups with a *p* value of *I* ^2^ < 0.05. The combined HRs of abnormal lncRNAs expression on OS in study cohort with qRT‐PCR and other methods were 2.00 (95% CI = 1.64–2.45, *p* < 0.001, fixed effect) and 0.99 (95% CI = 0.49–2.00, *p =* 0.981, random effect), respectively. Interestingly, studies with the qRT‐PCR method displayed no significant heterogeneity with *I* ^2^ = 32.4% and *p*(*I* ^2^) > 0.05, whereas studies with other methods to detect lncRNA expression had significant heterogeneity with *I* ^2^ = 94.7% and *P*(*I* ^2^) < 0.001. Table 4 shows that there was no significant heterogeneity in the subgroups analysis of univariate PFS (*I* ^2^ < 50%, *P*(*I* ^2^) > 0.05, fixed effect). These results suggested that different methods to assess lncRNA expression among studies may be a vital cause of heterogeneity in the univariate analysis of OS.

**TABLE 3:** Subgroup analysis of lncRNAs expression and OS in multiple myeloma patients

Subgroup analysis	No. of studies	No. of patients	Pooled HR (95% CI)	*p*	Heterogeneity		Model
					*I* ^2^ (%)	*p* value	
HR analysis method							
Univariate	23	3406	1.79 (1.38–2.32)	0.000	76.8%	0.000	Random
Multivariate	7	959	1.50 (1.16–1.95)	0.002	49.3%	0.055	Fixed
Ethnicity							
Asian	18	1657	2.01 (1.59‐2.55)	0.000	41.1%	0.032	Random
Caucasian	5	1749	1.32 (0.79–2.19)	0.289	89.3%	0.000	Random
Sample size							
<100	14	1017	1.93 (1.42–2.62)	0.000	47.9%	0.017	Random
≥100	9	2389	1.63 (1.09–2.44)	0.019	87.6%	0.000	Random
LncRNAs							
MALAT1	2	113	2.09 (1.16–3.75)	0.014	0	0.477	Fixed
TCF7	3	434	2.03 (1.50–2.74)	0.000	0	0.440	Fixed
NEAT1	3	249	2.32 (1.48–3.65)	0.000	0	0.523	Fixed
PVT1	2	332	1.18 (1.31–3.64)	0.003	0	0.772	Fixed
Maximum follow‐up time							
≥5 years	7	2194	1.88 (1.27–2.80)	0.002	58.9%	0.013	Random
<5 years	16	1212	1.74 (1.24–2.44)	0.001	81.2%	0.000	Random
ISS stage							
Stage I ≥35%	4	754	1.94 (0.75–5.04)	0.175	92.3%	0.000	Random
Stage I <35%	13	1511	1.69 (1.32–2.16)	0.000	44.5%	0.037	Random
Serotype IgG							
IgG >55%	7	1234	1.74 (0.98–3.07)	0.058	89.0%	0.000	Random
IgG ≤55%	7	481	1.57 (0.94–2.62)	0.088	67.7%	0.003	Random
Serotype IgA							
IgA >25%	4	238	1.86 (1.01–3.44)	0.049	59.4%	0.043	Random
IgA ≤25%	10	1477	1.57 (0.98–2.53)	0.062	86.7%	0.000	Random
Method							
qRT‐PCR	20	1811	2.00 (1.64–2.45)	0.000	32.4%	0.072	Fixed
Other	3	1596	0.99 (0.49–2.00)	0.981	94.7%	0.000	Random
Cutoff value							
Median	16	1811	1.79 (1.38–2.32)	0.000	49.3%	0.011	Random
Other	7	1595	1.91 (0.94–3.88)	0.075	86.9%	0.000	Random

Abbreviations: CI, confidence interval; HR, hazard ratio; IgG, immunoglobulin G; IgA, immunoglobulin A; ISS, International Staging System; lncRNA, long noncoding RNA; OS, overall survival; qRT‐PCR, quantitative reverse transcription‐polymerase chain reaction.

**TABLE 4:** Subgroup analysis of lncRNAs expression and PFS in multiple myeloma patients

Subgroup analysis	No. of studies	No. of patients	Pooled HR (95%CI)	*p*	Heterogeneity		Model
					*I* ^2^ (%)	*p* value	
HR analysis method							
Univariate	11	2079	1.40 (1.26–1.56)	0.000	30.8%	0.153	Fixed
Multivariate	4	747	1.82 (1.37–2.42)	0.000	49.2%	0.116	Fixed
Ethnicity							
Asian	9	1040	1.63 (1.40–1.90)	0.000	0	0.554	Fixed
Caucasian	2	1039	1.36 (1.07–1.43)	0.004	1.5%	0.314	Fixed
Sample size							
<100	5	378	1.96 (1.49–2.58)	0.000	0	0.868	Fixed
≥100	6	1701	1.33 (1.19–1.49)	0.000	23.8%	0.255	Fixed
LncRNAs							
PVT1	2	332	1.88 (1.30–2.72)	0.001	0	0.469	Fixed
MALAT1	2	122	2.02 (1.26–3.26)	0.004	0	0.329	Fixed
Maximum follow‐up time							
≥5 years	5	1406	1.32 (1.14–1.54)	0.000	39.0%	0.336	Fixed
<5 years	6	673	1.71 (1.36–2.14)	0.000	43.5%	0.248	Fixed
Serotype IgG							
IgG >55%	3	246	1.76 (1.31–2.37)	0.002	0	0.766	Fixed
IgG ≤55%	5	590	1.58 (1.31–1.90)	0.000	32.3%	0.206	Fixed
Serotype IgA							
IgA >25%	3	259	2.04 (1.46–2.86)	0.223	0	0.580	Fixed
IgA ≤25%	5	577	1.53 (1.24–1.83)	0.000	0	0.476	Fixed
Cutoff value							
Median	9	1211	1.45 (1.25–1.68)	0.000	31.0%	0.170	Fixed
Other	2	249	1.96 (1.10–3.49)	0.022	22.2%	0.257	Fixed
Method							
qRT‐PCR	9	1040	1.63 (1.40–1.90)	0.000	0	0.554	Fixed
Other	2	1039	1.24 (1.07–1.43)	0.004	1.5%	0.314	Fixed

Abbreviations: CI, confidence interval; HR, hazard ratio; IgG, immunoglobulin G; IgA, immunoglobulin A; ISS, International Staging System; lncRNA, long noncoding RNA; PFS, progression‐free survival; qRT‐PCR, quantitative reverse transcription‐polymerase chain reaction.

### 3.6 Sensitivity analysis and publication bias

**FIGURE 5:**
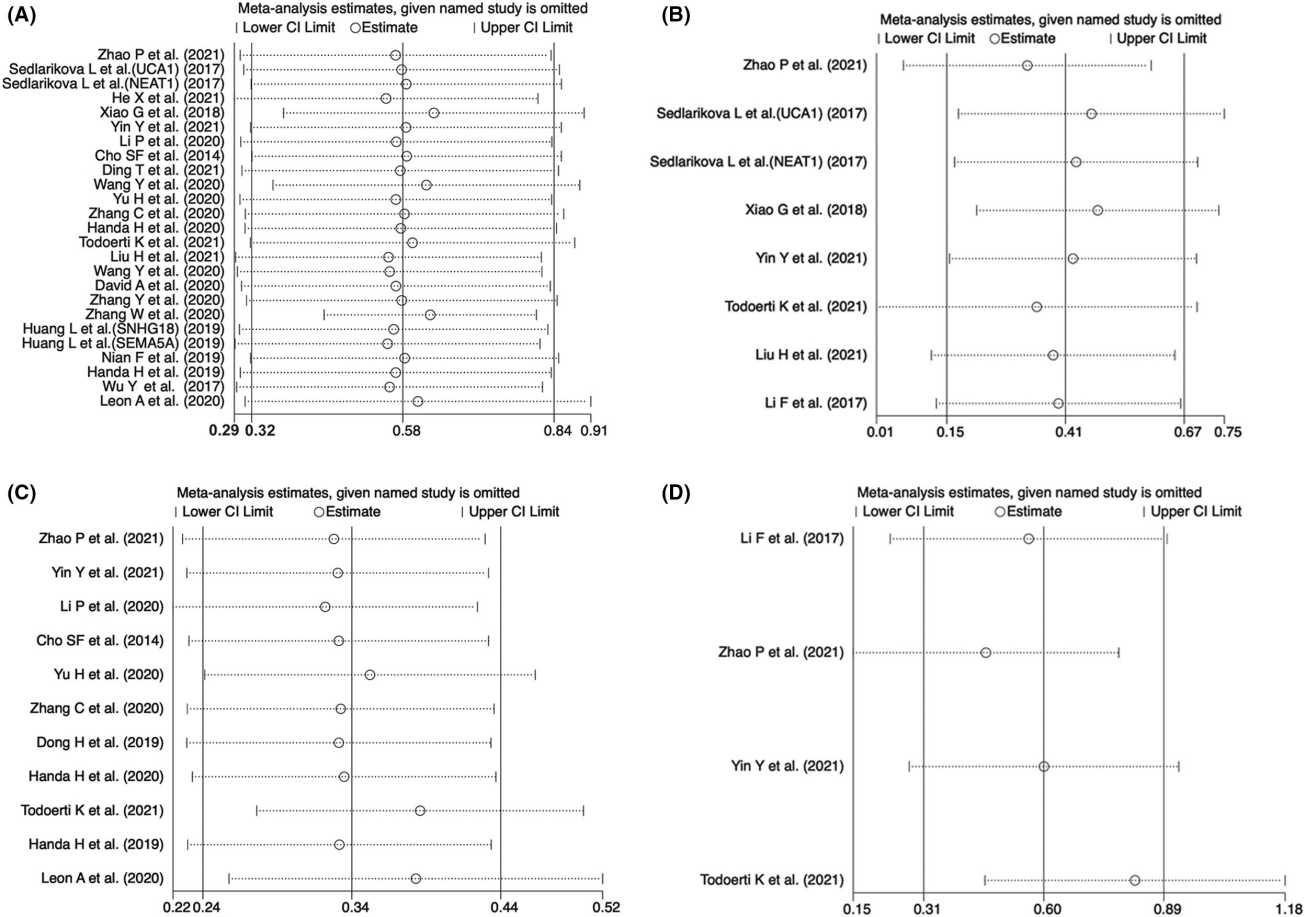
Sensitive analysis of meta‐analysis. (A) Sensitive analysis for univariate overall survival (OS), (B) sensitive analysis for multivariate OS, (C) sensitive analysis for univariate progression‐free survival (PFS), and (D) sensitive analysis for multivariate PFS.

**FIGURE 6:**
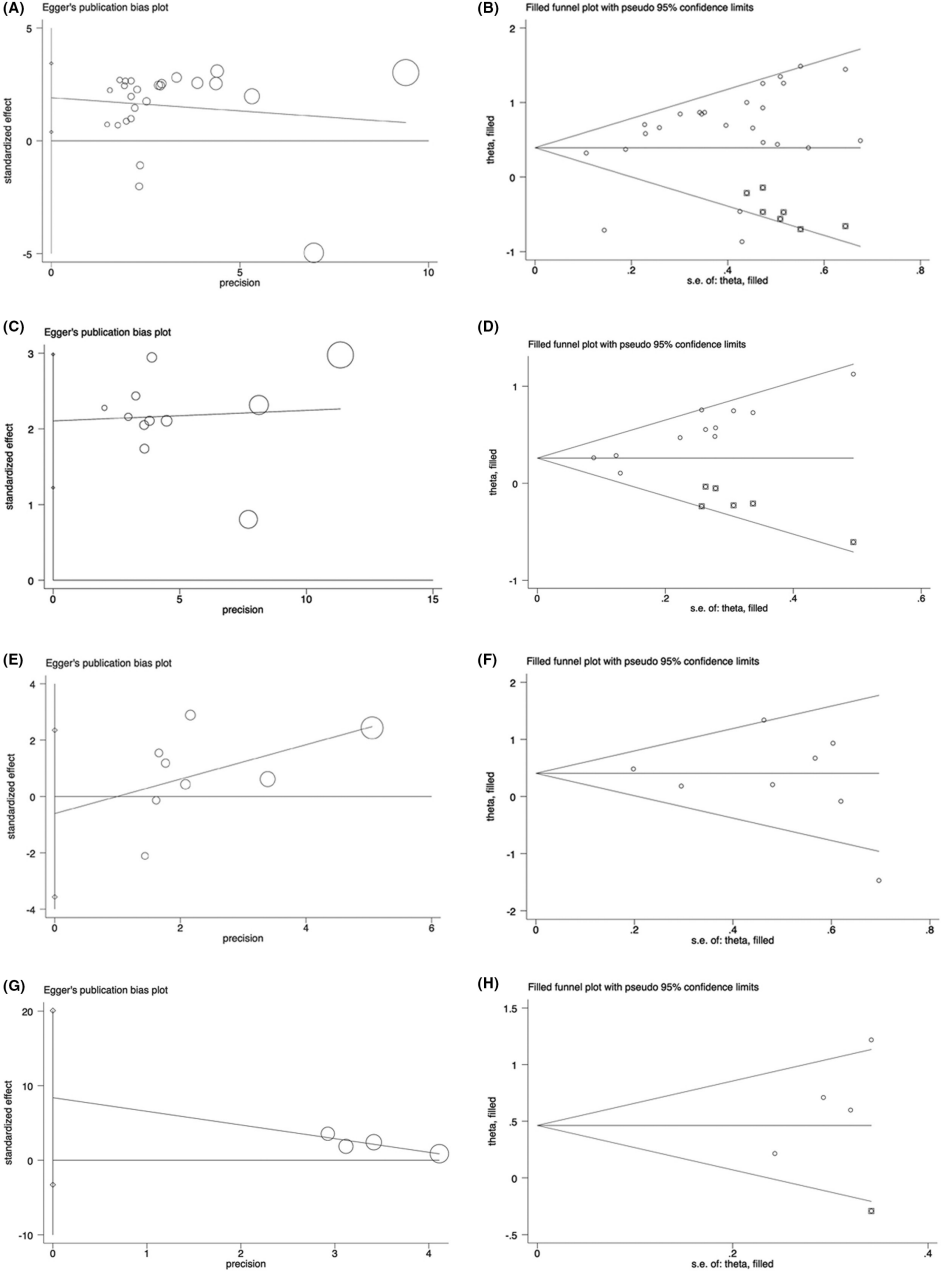
Egger's plot for publication bias analysis and trim and fill analysis for interpolation of potential unpublished studies. (A) Egger's plot and (B) trim and fill analysis based on univariate overall survival (OS), respectively, (C) Egger's plot and (D) trim and fill analysis based on univariate progression‐free survival (PFS), respectively, (E) Egger's plot and (F) trim and fill analysis based on multivariate OS, respectively, (G) Egger's plot and (H) trim and fill analysis based on multivariate PFS, respectively. For Egger's plot, the diameter of circles is presented as the weight of studies; for trim and fill analysis, 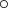 means included studies, whereas 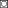 means estimated missing studies after adjustment for publication bias.

Sensitivity analysis was performed to conduct whether the relationship between abnormally expressed lncRNAs and OS or PFS was interfered with by individual studies. As shown in Figure 5, there was no significant change in the pooled results when any individual studies were removed in the univariate or multivariate OS and PFS, indicating the result was reliable and stable. Moreover, we used Begg's and Egger's tests to determine the publication bias, showing that there was no significant publication bias in multivariate OS or PFS (for multivariate OS, *p =* 0.386 for Begg's test and *p =* 0.634 for Egger's test; for multivariate PFS, *p =* 0.308 for Begg's test and *p =* 0.091 for Egger's test) (Table 5). However, a significant publication bias was observed in univariate OS and PFS (for univariate OS, *p =* 0.386 for Begg's test and *p =* 0.016 for Egger's test; for univariate PFS, *p =* 0.013 for Begg's test and *p* < 0.001 for Egger's test). Egger's funnel plot of the pooled analysis on the association between lncRNA and PFS or OS was also performed, and trim and fill analysis of Duval and Tweedie was used to evaluate the number of missing studies. We noticed significant publication bias based on the univariate analysis of OS and PFS studies (Figure 6A,C). Then, we recalculated the pooled odds ratio with the addition of those missing hypothetical studies and further plotted the filling funnel. Those circles with squares outside show some missing studies that lay in the plots of trim and fill analysis (Figure 6B,D). The univariate analysis of OS and PFS studies were added with 7 and 6 studies, respectively. The pooled HRs for univariate and multivariate OS were 1.482 (95% CI = 1.17–1.88, *p* < 0.001) and 1.501 (95% CI = 1.16–1.95, *p* < 0.001), respectively. Additionally, the pooled HRs for univariate and multivariate PFS were 1.296 (95% CI = 1.18–1.43, *p* < 0.001) and 1.589 (95% CI = 1.22–2.07, *p* < 0.001), respectively.

**TABLE 5:** The result of trim and fill analysis as well as Begg's and Egger's tests

Parameter	Overall survival		Progression‐free survival	
	Univariate	Multivariate	Univariate	Multivariate
Trim and fill analysis				
Effect model	Random	Fixed	Fixed	Fixed
No. of studies used to trim	7	0	6	1
No. of studies after trim	32	8	17	5
HR (fixed method)	—	1.501	1.296	1.589
95% CI (fixed method)	—	1.156–1.947	1.178–1.426	1.221–2.068
*p* for HR (fixed method)	—	0.002	0.000	0.001
*Z* (fixed method)	—	3.051	5.311	3.447
HR (random method)	1.482	—	—	—
95% CI (random method)	1.171–1.876	—	—	—
*p* for HR (random method)	0.001	—	—	—
*Z* (random method)	3.277	—	—	—
Begg's test				
Number of Studies	25	8	11	4
Adj.Kendall's Score	52	−8	33	4
Standard deviation	42.82	8.08	12.85	2.94
*Z* (continuity corrected)	1.19	0.87	2.49	1.02
*p* (continuity corrected)	0.234	0.386	0.013	0.308
Egger's test				
Number of studies	25	8	11	4
Coefficient	1.912	−0.606	2.103	8.397
95% CI of coefficient	0.391–3.432	−3.654–2.352	1.222–2.984	−3.28–20.07
*p*	0.016	0.634	0.000	0.091
Standard error	0.735	1.209	0.389	2.715
*t*	2.6	−0.5	5.4	3.09

Abbreviations: CI, confidence interval; HR, hazard ratio.

**FIGURE 7:**
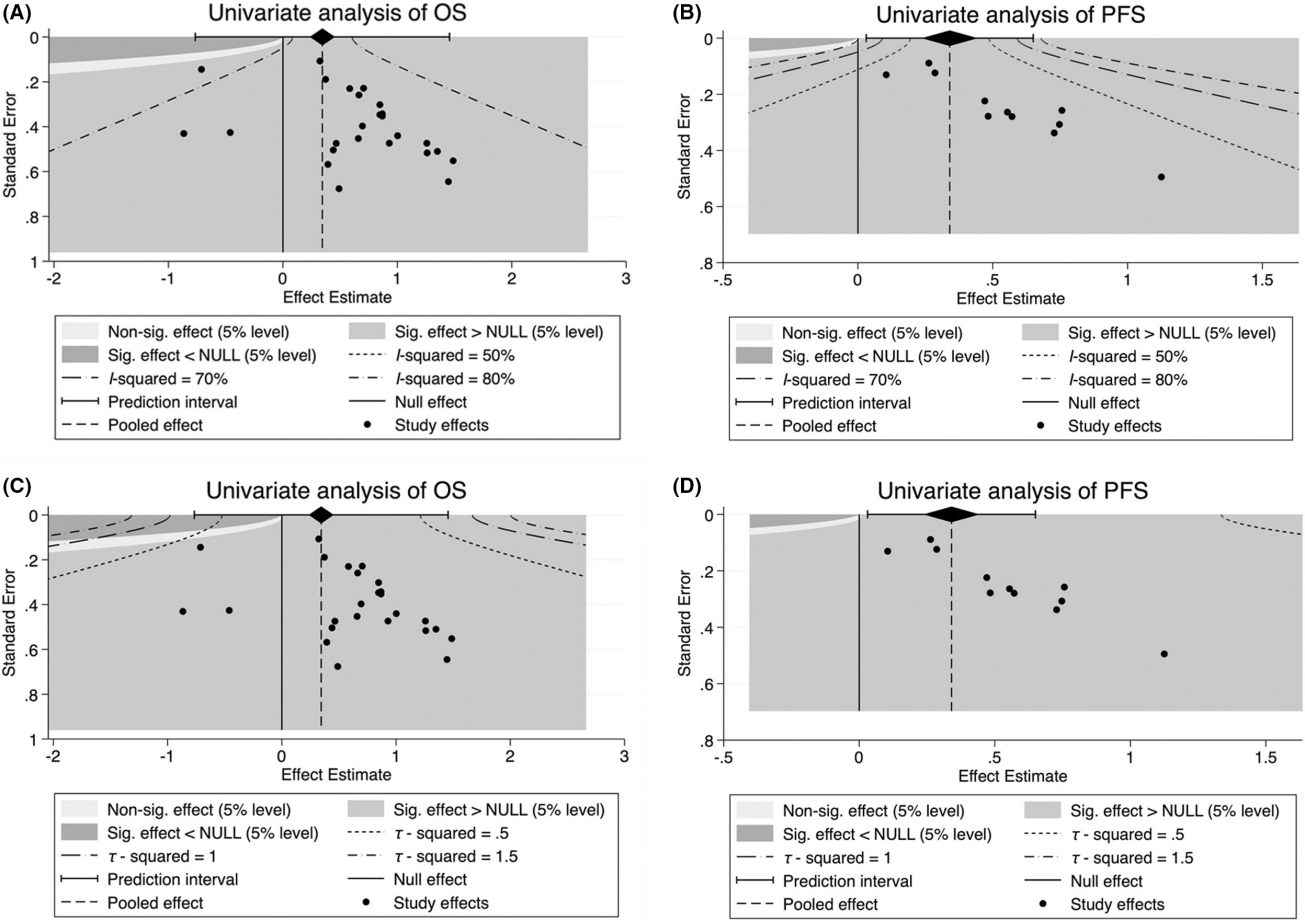
Contour‐enhanced funnel plot was used to predict the influence of publication bias. *I* ^2^ value based on univariate analysis of (A) overall survival (OS) and (B) progression‐free survival (PFS), respectively. *τ* ^2^ value based on univariate analysis of (C) OS and (D) PFS, respectively.

Finally, considering the significant publication bias in univariate OS and PFS, we performed a contour‐enhanced funnel plot to predict whether publication bias could weaken the reliability of our conclusion. As demonstrated in Figure 7A,B, “sig. effect > NULL” accounts for the major estimate area in both univariate analyses of OS and PFS, suggesting that the result of those unpublished articles will more likely support our conclusions. Furthermore, we evaluated the *I* ^2^ and *τ* ^2^ values of publication bias, indicating that the *I* ^2^ value was less than 80%, whereas the *τ* ^2^ of total studies were <0.5 in univariate analysis of OS (Figure 7A,C). In addition, the *I* ^2^ and τ^2^ values of univariate analysis of PFS were <50% and <0.5, respectively (Figure 7B,D). These results suggest the reliability of our current conclusion despite publication bias exists.

### 3.7 Biological features of abnormally expressed lncRNAs

Furthermore, we scrutinized all the included lncRNAs and summarized the details of location, function, targets, and the expression level, the corresponding data are shown in Table 6. These abnormally expressed lncRNAs located in different chromosome positions and were involved in cell proliferation and apoptosis regulation. Some of these lncRNAs affected cell metabolism (e.g., high expression of MALAT1‐accelerated glycolysis), whereas some lncRNAs influenced myeloma cell invasion. PCAT1, ANRIL, H19, ANGPTL1‐3, or NEAT1 upregulation induced myeloma cells insensitive to bortezomib, whereas CRNDE overexpression leads to dexamethasone tolerance.

**TABLE 6:** Function and targets of long noncoding RNAs in multiple myeloma

Symbol	Location	Function	Target	Abnormal expression^a^	Prognosis	Reference (PMID)
PCAT1	08q24.21	∆ PCAT1: ↑cell division,↓apoptosis, ↓sensitivity to bortezomib	miR‐129	Upregulation	Poor	31777580
UCA1	19p13.12	∆ UCA1: ↑cell division, ↓apoptosis	miR‐331‐3p, miR‐1271‐5p	Upregulation	Poor	31773675
PRAL	17p13.1	∆ PRAL: ↓cell division, ↑apoptosis	miR‐210	Downregulation	Poor	29944867
ANRIL	09p21.3	∆ ANRIL:↑cell division,↓apoptosis, ↓sensitivity to bortezomib	miR‐34a, miR‐125a, miR‐186, PTEN	Upregulation	Poor	33528317 34034498
PVT1	08q24.21	∆ PVT1:↑cell division,↓apoptosis	miR‐486, miR‐203a	Upregulation	Poor	31900844
MALAT1	11q13.1	∆ MALAT1:↑cell division,↓apoptosis, ↑glycolysis	miR‐1271‐5p, miR‐1271‐5p, miR‐181a‐5p	Upregulation	Poor	31397203 31953613
TCF7	05q31.1	∆ TCF7:↑cell division,↓apoptosis	miR‐200c, miR‐203	Upregulation	Poor	32578294
OIP5‐AS1	15q15.1	∆ OIP5‐AS1:↓cell division,↑apoptosis, ↓viability, ↓invasion	miR‐27a‐3p, miR‐3163	Downregulation	Poor	32410883 32329664
NEAT1	11q13.1	∆ NEAT1:↑cell division,↓apoptosis, ↓sensitivity to bortezomib	miR‐215a, miR‐29b‐3p	Upregulation	Poor	32608537 33253679
ST3GAL6‐AS1	03q12.1	∆ ST3GAL6‐AS1:↑cell division,↓apoptosis	hnRNPA2B1	Upregulation	Poor	33649796
LINC01606	08q12.1	∆ LINC01606:↑cell division,↓apoptosis, ↑viability, ↑invasion	miR‐579‐3p	Upregulation	Poor	34539994
SNHG6	08q13.1	∆ SNHG6:↑cell division,↓apoptosis	Unknown	Upregulation	Poor	34638381
CRNDE	16q12.2	∆ CRNDE:↑cell division,↓apoptosis, ↑invasion, ↓sensitivity to dexamethasone	miR‐451, IL6R	Upregulation	Poor	28276319 32879426
H19	11p15.5	∆ H19:↑cell division,↓apoptosis, ↑colony formation,↓sensitivity to bortezomib	miR‐152‐3p,miR‐29b‐3p, Akt	Upregulation	Poor	33040789 31712391
ANGPTL1‐3	01q25.2	∆ ANGPTL1‐3:↑cell division,↓apoptosis,↓sensitivity to bortezomib	miR‐30a‐3p	Upregulation	Poor	31103265
SNHG18	05p15.3	∆ SNHG18:↑cell division,↓apoptosis, ↑viability, ↑invasion	miR‐211‐5p	Upregulation	Poor	33500406
SEMA5A	05p15.3	∆ SEMA5A:↑cell division,↓apoptosis, ↑viability, ↑invasion	miR‐204	Upregulation	Poor	30454024
EPB41L4A	05q22.1	∆EPB41L4A: ↓cell division, ↑apoptosis, ↓viability, ↓invasion	Unknown	Downregulation	Poor	33193600 35181612
TUG1	22q12.2	∆ TUG1:↑cell division,↓apoptosis, ↑viability, ↑invasion	miR‐29b‐3p	Upregulation	Poor	30842339
PDLIM1P4	03q12.1	Unknown	Unknown	Upregulation	Poor	33597729

^a^ Expression value in MM vs control group; ∆ upregulation.

## 4 DISCUSSION

Multiple myeloma is a common hematological disease and ranks second in terms of plasma cell malignancies worldwide.^1^ The condition has a high heterogeneity with prognoses that varies widely across subgroups. Although the ISS stage has been extensively used in clinical therapeutic strategies, patients within similar ISS stage groups may present heterogeneous prognostic features. Therefore, other variables are necessary to complement the ISS system to stratify patients with high‐risk factors. LncRNAs are actively transcribed genes that do not code proteins and have a minimum transcript length of 200 bp. Numerous lncRNAs have been recognized as crucial regulators in multiple cellular processes,^54^ including the cell cycle,^21^, ^55^ apoptosis,^13^ and metabolism,^56^, ^57^ as well as in tumorigenesis.^20^ Multiple lncRNAs have also been identified to be implicated with poor prognosis in breast cancer,^58^ colon cancer,^59^ gastric cancer,^60^ and bladder cancer.^61^, ^62^ Although accumulating studies have investigated the predictive value of abnormally expressed lncRNA levels in MM, its role was inconsistent and inclusive. Herein, we retrospected the published studies and performed a meta‐analysis to better understand the predictive value of abnormally expressed lncRNAs in MM.

Our meta‐analysis covered 3501 MM patients from different research centers, showing that abnormally expressed lncRNAs significantly predict poor OS (for univariate, HR = 1.79, 95% CI = 1.38–2.32, *p* < 0.001; for multivariate, HR = 1.50, 95% CI = 1.16–1.95, *p* = 0.055) and PFS (for univariate, HR = 1.40, 95% CI = 1.26–1.56, *p* < 0.001; for multivariate, HR = 1.82, 95% CI = 1.37–2.42, *p* < 0.001). Univariate analysis of OS displayed significant heterogeneity, and subgroup analysis suggested that different methods to assess lncRNA expression among studies may be the vital source of heterogeneity in univariate analysis of OS. Meanwhile, Begg's and Egger's tests were performed to correct publication bias, showing that the pooled HRs for univariate and multivariate OS were 1.48 (95% CI = 1.17–1.88, *p* < 0.001) and 1.50 (95% CI = 1.16–1.95, *p* < 0.001), respectively. Additionally, the pooled HRs for univariate and multivariate PFS were 1.30 (95% CI = 1.18–1.43, *p* < 0.001) and 1.59 (95% CI = 1.22–2.07, *p* < 0.001), respectively. Furthermore, we evaluated the heterogeneity of our studies based on contour‐enhanced funnel plots, demonstrating that “Sig. effect > NULL” accounts for the major estimate area, and our conclusion is relatively reliable despite publication bias exists.

Previous studies have demonstrated that TCF7 can be used to independently predict worse OS in epithelial ovarian cancer,^63^ nonsmall lung cancer,^64^ and colorectal cancer.^65^ Several reviews have summarized that NEAT1,^66^ MALAT1^67^ or PVT1^68^, ^69^ leads to carcinogenesis in multiple types of carcinoma. In our study, subgroup analysis suggested that upregulation of MALAT1, TCF7, NEAT1, and PVT1 were associated with poor OS (*p* < 0.05), high expression of PVT1, and TCF7 resulted in worse PFS (*p* < 0.05), while only overexpression of TCF7 was correlated with poor EFS (*p* < 0.05), indicating the predictive ability of particular lncRNAs in predicting prognosis of MM.

Emerging as regulators of diverse biological processes, lncRNAs have attracted much attention focusing on how aberrant lncRNAs expression lead to the poor prognosis of MM.^17^, ^20^, ^39^, ^41^, ^42^ On one hand, lncRNAs transcription initiates at a specific time and location corresponding to different stressors, which may result in chromosome abnormalities. For instance, the expression of lncRNA TCF7 is positively correlated with t(4, 14) and del(17p) in MM patients.^23^ LncRNA MALAT1 is located at 11q13.1 and is involved in regulating different signaling pathways, including MAPK/ERK, PI3K/AKT, and β‐catenin/Wnt through its targets.^20^, ^21^ Chromatin abnormalities are often accompanied by this process, and targeting MALAT1 may be an effective therapeutic strategy for MM.^70^ On the other hand, lncRNAs regulate cell proliferation and apoptosis through binding specific miRNAs. PRAL inhibits cell proliferation and induces apoptosis by targeting miR‐210 and further improves myeloma cells’ sensitivity to bortezomib.^17^ LncRNA TCF7 promotes cell proliferation and reduces cell apoptosis through downregulating miR‐200c in myeloma cells.^24^ Furthermore, lncRNA can impact tumor features by regulating RNA‐mediated signaling pathways or the expression level of other RNAs. LncRNA NR_046683, known as ST3GAL6‐AS1, is markedly upregulated in MM patients, which accelerates myeloma cell invasion by suppressing hnRNA2B1‐mediated ST3GAL6 expression. In contrast, knockdown of ST3GAL6‐AS1 suppresses the adhesion, migration, and invasion ability of MM cells in vitro.^71^ Interestingly, lncRNA NEAT1 interacts with miR‐29b‐3p, forming a positive feedback loop, thereby modulating bortezomib resistance,^72^ but it may also combine with microRNA‐125a to accelerate cell division and inhibit apoptosis.^38^ Meanwhile, various lncRNAs directly target other functional genes, such as oncogenes, as regulatory mechanisms. PVT1 shows three major molecular mechanisms: DNA rearrangement, microRNA encoding, and MYC interaction.^73^ Copy number of PVT1 is increased in more than 98% of MYC‐copy‐accumulate tumors.^74^ A study has demonstrated the positive correlation between PVT1 and MYC, and the high expression of PVT1 and MYC are correlated with MM progression.^14^ OIP5‐AS1 suppression facilitates cell proliferation and inhibits apoptosis by directly targeting KLF10 via activating the PTEN/PI3K/AKT pathway in myeloma cells.^37^, ^75^, ^76^ Distinguished as a valuable lncRNA for hematological malignancies therapy, CRNDE participates in multiple biological processes, such as cell proliferation, differentiation, migration, and apoptosis.^77^ CRNDE locus deletion decreases proliferation and increases sensitivity to dexamethasone of MM cells by impairing the IL6 signaling pathway.^44^ LncRNA H19s, a novel therapeutic target, regulates multiple oncogenic signaling pathways, including PI3K/Akt, canonical Wnt/β‐catenin, canonical NF‐κB, MAPK, and JAK/STAT pathway.^78^ It promotes tumorigenesis and malignant progression through sequestering some miRNAs, such as let‐7c, miR‐22‐3p, and miR‐675.^79^ In addition, H19 knockdown suppresses MM tumorigenesis by inhibiting cell proliferation through targeting miR‐152‐3p.^80^ Meanwhile, high expression of H19 leads to vincristine resistance in MM patients, in turn leading to poor prognosis.^50^ SNHG18 promotes cell proliferation and metastasis in glioma^81^ and nonsmall‐cell lung cancer.^82^ It is also associated with poor prognosis in MM,^47^ but the underlying mechanism remains unclear. TUG1, a pivotal oncogenic lncRNA, is aberrant upregulation among different types of cancer. It has been proposed that TUG1 knockdown suppresses cell proliferation, invasion, or colony formation^83^ and impedes the tumorigenesis of MM by regulating microRNA‐34a‐5p expression.^53^ As suggested by Ronchetti et al., lnc‐ANGPTL1‐3 was significantly upregulated in MM patients,^25^ which was associated with poor prognosis and activated c‐Maf expression via sponging miR‐30a‐3p, thereby inhibiting the effects of proteasome inhibitors.^48^ Collectively, aberrantly expressed lncRNAs are involved in the mechanisms of tumorigenesis of myeloma. Herein, we propose that the lncRNAs may be promising prognostic indicators and novel targets for the clinical treatment of MM patients.

Despite our efforts to improve this study, several limitations could not be ignored. First, our research is a literature‐based analysis, and possible publication bias may be somewhat ascribed to the inclination for positive results. However, we performed trim and fill analysis to correct potential publication bias and further performed a contour‐enhanced funnel plot to predict whether the publication bias of included studies would weaken the reliability of our conclusion. We noticed that our conclusion is relatively reliable despite publication bias exists. Second, only four of the included studies provided multivariate PFS clinical data. Thus, we fail to figure out the comprehensive heterogeneous factors based on the multivariate analysis of PFS. Third, we admit that different methods to assess lncRNA expression among studies may lead to heterogeneity in univariate analysis of OS, and different cutoff values may also result in heterogeneity, which need to be further investigated based on more available studies. Fourth, since the deficiency of HRs and 95% CI in several studies, we calculated a few univariate HRs from the Kaplan–Meier survival curves, which might be inconsistent with the original statistics. Fifth, some clinical data, such as ISS stage and serotype, are missing in a few studies, so subgroup pooled HRs in these criteria are limited.

In summary, our study uncovers that abnormally expressed lncRNAs have significant unfavorable impacts on the prognosis of MM patients, which might be used as potential novel prognostic molecular markers. However, different cutoff values and dissimilar methods to assess lncRNA expression among studies may lead to heterogeneity. The effect of lncRNAs in MM deserved more clinical investigations, performing prospective large‐population cohorts, to identify their prognostic role in treatment strategy‐making and lncRNA‐based therapy.

## AUTHORS' CONTRIBUTION

JDQ wrote the original manuscript. JDQ and BK carried out conceptualization, literature review, data collection, statistical analysis, writing—review and editing. TTL, HF, and ANL carried out methodology, literature review, data collection, and statistical analysis. CFK and TTL verified the data and analytical methods. CHJ was involved in conceptualization and made contributions to the design of the work. All the authors read and approved the final manuscript.

## FUNDING INFORMATION

This work was supported by the Translational Research Grant of NCRCH (No. 2021WWA02), the Central Guidance of Local Science and Technology Development Fund (No.20211ZDG02002), National Natural Science Foundation of China (No.81560026), Key R & D plan of Jiangxi Province of China (No. 20202BBGL73111).

## CONFLICT OF INTEREST

The authors declare that they have no conflict of interest.

## ETHICAL APPROVAL

This article does not contain any studies with human participants or animals performed by any authors.

## Data Availability

The data and materials analyzed in our study are available from the corresponding author upon reasonable request.
